# Is Prehospital Time Important for the Treatment of Severely Injured Patients? A Matched-Triplet Analysis of 13,851 Patients from the TraumaRegister DGU®

**DOI:** 10.1155/2019/5936345

**Published:** 2019-06-20

**Authors:** Konstantin Klein, Rolf Lefering, Pascal Jungbluth, Sven Lendemans, Bjoern Hussmann

**Affiliations:** ^1^Department of Trauma and Hand Surgery, University Hospital, Moorenstrasse 5, 40225 Duesseldorf, Germany; ^2^Institute for Research in Operative Medicine (IFOM), Witten/Herdecke University, Ostmerheimer Str. 200, 51109 Cologne, Germany; ^3^Department of Trauma Surgery, Alfried Krupp Hospital, Alfried-Krupp-Strasse 21, 45131 Essen, Germany; ^4^University of Duisburg-Essen, Germany

## Abstract

**Background:**

The impact of time (the golden period of trauma) on the outcome of severely injured patients has been well known for a long time. While the duration of the prehospital phase has changed only slightly (average time: ~66 min) since the TraumaRegister DGU® (TR-DGU®) was implemented, mortality rates have decreased within the last 20 years. This study analyzed the influence of prehospital time on the outcome of trauma patients in a matched-triplet analysis.

**Material and Methods:**

A total of 93,024 patients from the TraumaRegister DGU® were selected based on the following inclusion criteria: ISS ≥ 16, primary admission, age ≥ 16 years, and data were available for the following variables: prehospital intubation, blood pressure, mode of transportation, and age. The patients were assigned to one of three groups: group 1: 10-50 min (short emergency treatment time); group 2: 51-75 min (intermediate emergency treatment time); group 3: >75 min (long emergency treatment time). A matched-triplet analysis was conducted; matching was based on the following criteria: intubation at the accident site, rescue resources, Abbreviated Injury Scale (AIS) of the body regions, systolic blood pressure, year of the accident, and age.

**Results:**

A total of 4,617 patients per group could be matched. The number of patients with a GCS score ≤8 was significantly higher in the first group (group 1: 36.6%, group 2: 33.5%, group 3: 30.3%; p < 0.001). Moreover, the number of patients who had to be resuscitated during the prehospital phase and/or upon arrival at the hospital was higher in group 1 (p = 0.010); these patients also had a significantly higher mortality (group 1: 20.4%, group 2: 18.1%, group 3: 15.9%; p ≤ 0.001). The number of measures performed during the prehospital phase (e.g., chest tube insertion) increased with treatment time.

**Conclusions:**

The results suggest that survival after severe trauma is not only a matter of short rescue time but more a matter of well-used rescue time including performance of vital measures already in the prehospital setting. This also includes that rescue teams identify the severity of injuries more rapidly in the most-severely injured patients in critical condition than in less-severely injured patients and plan their interventions accordingly.

## 1. Introduction

The prehospital phase is still crucial for the outcomes of severely injured patients. In particular, the term “the golden period of trauma” is of considerable importance in this context [1, 2]. With regard to the golden period of trauma, a paradigm shift has occurred, particularly in German-speaking countries. While in the early 1990s management was aimed at comprehensive therapy at the accident site, currently, the strategy is to stabilize trauma patients at the site of the accident and transfer them to the hospital as soon as possible. Unless it is essential for patient survival, medical treatment should not be performed at the accident site [3–5]. This treatment regime is based on a US study among patients with severe abdominal injuries conducted by Clarke et al., which provided evidence that mortality increases by 1% every 3 minutes [6]. Therefore, it has been postulated in Germany that, in most-severely injured patients, definitive therapy should be initiated within 90 minutes after initial injury. Accordingly, the time between the emergency call and the patient's admission to the target hospital should not exceed one hour [7]. Previous studies by our working group demonstrated a relationship between increased prehospital volume administration and the prolongation of emergency treatment time. It has been shown that these factors correspond to negative overall patient outcomes [8, 9]. Geraedts et al. confirmed this correlation based on a separate study [3]. During the late 1990s and the beginning of the current century, the US researcher Bickell demonstrated that improved outcomes are associated with short emergency treatment times and the fastest possible access to definitive therapy in a hospital setting, particularly in patients suffering penetrating trauma [10–12]. It is crucial not only to identify adverse events and errors occurring within these processes but also to identify errors and deaths that might have been prevented [13–15]. Davis et al. demonstrated that up to 6% of trauma-related deaths might have been prevented [16]. Gruen et al. identified delayed treatment of active torso hemorrhage (thorax, abdomen, and pelvis) as the most frequent cause of preventable deaths [15]. The delayed diagnosis and treatment of pelvic hemorrhages seems to be the number one “killer”. According to Soreide et al., this type of bleeding—except for traumatic brain injuries—causes most of the deaths, particularly within the early posttraumatic phase. Therefore, it is mandatory to improve the treatment of such bleeding [17]. This is also true with regard to the paramount importance of structured and meaningful transfusion and coagulation management, and multiple improvements are expected in the future [18].

The current report of the TraumaRegister DGU® shows that mortality has continuously decreased over the last 20 years. However, the average emergency treatment time of approximately 66 minutes has not changed since 1993 [2018 annual report of the TraumaRegister DGU®]. Hence, according to these data, hospital treatment within the “golden hour of shock” is still an illusion. Currently, there are no definitive evidence-based recommendations concerning prehospital treatment procedures with regard to emergency treatment time. In contrast, Osterwalder demonstrated in his Swiss study that the extension of the “golden hour of shock” is associated with improved outcomes in patients suffering blunt trauma [19].

A comprehensive review of the current literature suggested several unresolved issues concerning the major determining factors for the outcome of severe injuries. Of particular interest is whether emergency treatment time has an essential influence on the patient's outcome with regard to multiple organ failure (MOF), sepsis, and mortality. This study investigated these questions based on patients within the TraumaRegister DGU® who had suffered severe injuries (Abbreviated Injury Scale [AIS] >3).

## 2. Material and Methods

The TraumaRegister DGU® of the German Trauma Society (Deutsche Gesellschaft für Unfallchirurgie, DGU) was founded in 1993. The aim of this multicenter database is to provide anonymized and standardized documentation of severely injured patients.

The data are collected prospectively in four consecutive time phases from the site of the accident until discharge from the hospital: (A) prehospital phase, (B) emergency room and initial surgery, (C) intensive care unit, and (D) discharge. The documentation includes detailed information on demographics, injury pattern, comorbidities, pre- and in-hospital management, progression in the intensive care unit, and relevant laboratory findings, including data on transfusion and the outcome of each individual patient. The inclusion criterion is hospital admission via the emergency room with subsequent ICU/ICM or arriving at the hospital with vital signs and death before admission to the ICU. The infrastructure for documentation, data management, and data analysis is provided by the AUC, the Academy for Trauma Surgery (AUC, Akademie der Unfallchirurgie GmbH), a company affiliated with the German Trauma Society. The scientific leadership is provided by the Committee on Emergency Medicine, Intensive Care and Trauma Management (Sektion NIS) of the German Trauma Society. The participating hospitals submit their data anonymously into a central database via a web-based application. Scientific data analysis is approved according to a peer review procedure established by Sektion NIS. The participating hospitals are primarily located in Germany (90%), but an increasing number of hospitals in other countries contribute data as well (such as Austria, Belgium, China, Finland, Luxembourg, Slovenia, Switzerland, the Netherlands, and the United Arab Emirates). Currently, approximately 25,000 cases from more than 600 hospitals have been entered into the database per year. Participation in the TraumaRegister DGU® is voluntary. For hospitals associated with the TraumaNetzwerk DGU®, however, the entry of at least one basic data set is obligatory for reasons of quality assurance (as per previously described in [20, 21]).

The present study is in line with the publication guidelines of the TraumaRegister DGU® (TR-DGU) and is registered under the TR-DGU project ID M 2012-062. The study has the full approval by the local ethical committee (18-8089-BO).

The following patients met the criteria for matching:

1. Patients from Germany and Austria (to minimize variations caused by the utilization of different rescue systems).
2. Patients who were attended to by a physician prior to hospital admission.
3. Patients with primary admission to the hospital (no transfers).
4. Patients with an Injury Severity Score (ISS) ≥16.
5. Patients aged ≥16 years.
6. Patients with blunt trauma.
7. Patients with systolic blood pressure at the site of the accident >40 mm Hg.
8. Patients with data available for the following parameters: prehospital rescue time, blood pressure at the accident site, intubation, rescue resources, and age.

**Figure 1:**
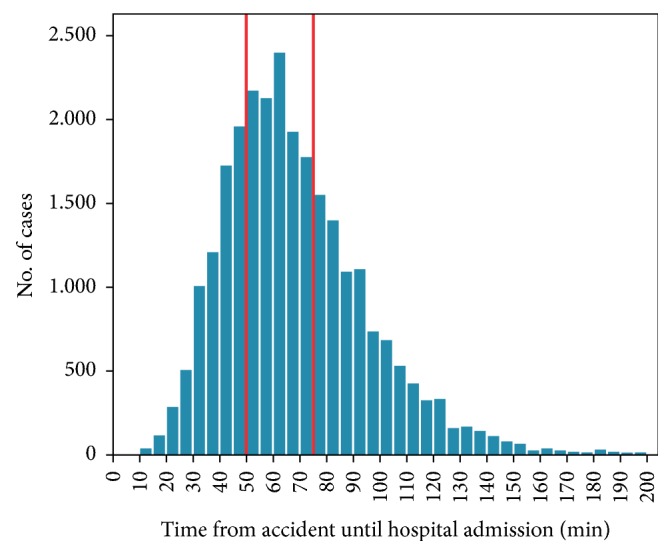
Mean value of prehospital time of all severely injured patients.

According to the prehospital emergency treatment time, patients were allocated to the following groups: group 1: short emergency treatment time (10-50 min), group 2: intermediate emergency treatment time (51-75 min), and group 3: long emergency treatment time (≥76 min). This classification was chosen since the mean duration of prehospital time in the TraumaRegister DGU® was approximately 70 minutes (Figure 1).

**Figure 2:**
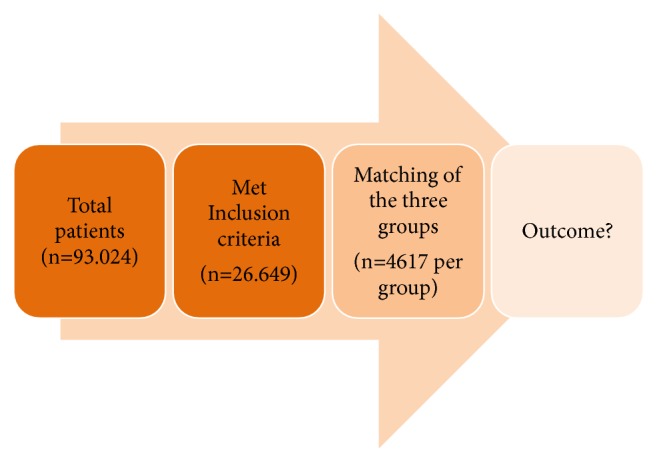
Graphic representation of test procedure.

To evaluate the influence of prehospital emergency treatment time, patients in the three groups were matched according to the following criteria (Figure 2):

- Injury pattern for five different body regions: head, thorax, abdomen, and extremities, including the pelvis, where the matching criterion was an “Abbreviated Injury Scale” (AIS) score of ≥ or < 3 points.
- To account for treatment changes that may have been established over the years, the date of injury was divided into four subgroups: (1) 1993-2001, (2) 2002-2005, (3) 2006-2008, and (4) 2009-2011.
- Blood pressure was subdivided into three groups with the following ranges: (1) 40-89 mm Hg, (2) 90-99 mm Hg. and (3) ≥100 mm Hg.
- Age was categorized into three subgroups: (1) 16-54, (2) 55-69, and (3) ≥70 years.
- Prehospital intubation (yes/no).
- Mode of transportation to the hospital (air vs. ground).

The selection of these parameters for the matched-pair analysis was aiming to result in statistically comparable groups with regard to the questioning, in order to make sure that relevant parameters influencing rescue time (e.g., preclinical intubation) have been distributed evenly across all groups. The same applies, e.g., to age as a potential influencing factor for outcome per se, or the severity of injuries of each body region. The selection was based on recently published studies with identical methodology [22].

Analysis of the emergency time (from arrival of the emergency physician to the time spent at the accident site to hospital admission) shows that the total emergency time is actually composed of all the different phases (group 1: 40 min; group 2: 62 min.; group 3: 101 min; p ≤ 0.001) and that the time spent at the accident site does not exclusively contribute to the time differences, although it represents the major part of total rescue time.

Sepsis criteria defined by the American College of Chest Physicians/Society of Critical Care Medicine (ACCP-SCCM) consensus conference were applied to verify the presence of sepsis [23]. Single organ failure (SOF) was defined using the Sequential Organ Failure Assessment (SOFA) score. A SOFA score of 3 or more represented a case of SOF [24].

The SOFA score was entered as the total value into the TraumaRegister DGU®; therefore, no conclusions could be drawn about management or interventions in individual patients. Simultaneous failure of two or more organs defined a case of multiple organ failure (MOF). Prehospital parameters, coagulation status, and length of hospital stay were investigated separately within each group. Coagulation status was determined by the use of the prothrombin ratio, which corresponds to the International Normalized Ratio (INR) and is a commonly used parameter in Germany.

To evaluate the ISS within groups with sufficiently complete data, prognosis estimation by means of the Revised Injury Severity Classification (RISC) was performed [25]. The prognosis was thus compared to the observed mortality rate within the corresponding group. Prognoses were also calculated according to the Trauma and Injury Severity Score (TRISS). Prediction of mass transfusion was analyzed using the Trauma Associated Severe Hemorrhage Score (TASH).

### 2.1. Statistics

Analysis was carried out using the Statistical Package for the Social Sciences (SPSS®; version 17, Chicago, IL, USA). Incidences are expressed as the numbers of cases and the percentages. Continuous variables are presented as the mean values with standard deviations (SD). Differences between the three matched groups were evaluated using the chi-square test for categorical variables and the analysis of variance (ANOVA) for continuous variables. In cases of obvious deviations from normality, a nonparametric rank test (Kruskal-Wallis) was performed to test continuous variables. A p-value <0.05 was considered statistically significant, although the large number of matched triplets would reveal significant results even in the case of minor differences. Therefore, statistical significance should always be considered together with the clinical relevance of the observed difference. This is particularly valid for continuous measurements where differences of 0.04 *∗* SD (one twenty-fifth of a standard deviation) would be statistically significant because of the large sample size.

## 3. Results

A total of 4,617 severely injured patients in group 1 were matched with 4,617 patients in group 2 and with 4,617 patients in group 3. The average age in the overall patient population was 45.6 years. In comparison, the largest proportion of male patients was found in group 2 (group 1: 71.8%, group 2: 74.2%, group 3: 72.4; p = 0.030). The ISS was almost identical in the three groups (group 1: 29.2; group 2: 28.6; group 3: 28.2; p = 0.100). The distribution of injury severity levels in the corresponding body regions was similar across the groups (Table 1). With 62.6%, traffic accidents were the most common trauma mechanism. No significant differences between the groups were observed. The similarity of the general characteristics within the three groups supports the assumption of valid comparability among patients with short, intermediate, and long emergency treatment times. Particularly in terms of the initially determined matching parameters, one can summarize—as shown in Tables 1 and 2(c)—that those results did* not* show statistically significant differences, i.e., were statistically identical (e.g., Table 1; AIS Thorax ≥3 with a p-value of 1), and, thus, that the matching process has been successful allowing for further analyses.

### 3.1. Prehospital and Emergency Department Treatment

As shown in Table 2(a), there was no difference between the three groups when comparing systolic blood pressures during the prehospital phase and at the time of hospital admission. However, the percentage of patients with shock (BP ≤90 mmHg) upon arrival in the hospital was highest in group 1, and the difference was significant (group 1: 14.7%; group 2: 14.1%; group 3: 12.6%, p = 0.010).

Hemoglobin concentration, base excess, and coagulation values (prothrombin ratio, prothrombin time) were determined within the emergency department (Table 2(b)). The prothrombin ratio was higher in group 1 than in group 2 or 3 (group 1: 80.7%; group 2: 80.0%; group 3: 79.6%, p = 0.030). There was no difference with regard to the administration of blood products such as packed red blood cells and fresh frozen plasma (Table 2(c)).

The number of prehospital measures (chest tubes, sedation) has been observed to increase with increasing emergency treatment time (Table 2(c)). With regard to chest tubes, we observed a significantly larger number of patients who had chest tubes inserted in group 3 compared with the other groups (Table 2(c)). The prehospital administered volume increased in proportion to the prehospital time when compared across the three groups (group 1: 1,135 ml; group 2: 1,280 ml; group 3: 1,461 ml, p ≤ 0.001). The percentage of patients resuscitated in a prehospital setting was highest in group 1 (group 1: 5.2%; group 2: 4%; group 3: 3%; p ≤ 0.001). Similar results were observed with regard to patients who required resuscitation at arrival in the hospital (Table 2(c)).

### 3.2. Clinical Course and Outcome

The number of patients treated with early surgical therapy was different between the three groups (group 1: 6.7%; group 2: 5.8%; group 3: 5.2%; p = 0.02, Table 3). The time spent in the intensive care unit (ICU), the length of the hospital stay, and the total days of intubation were similar in the three groups (Table 3). The occurrence of sepsis and organ failure did not differ significantly between the three groups (Table 3). The occurrence of multiple organ failure was highest in group 1, and the difference was significant (group 1: 31.6%; group 2: 29.6%; group 3: 28.2%; p = 0.01).

The TRISS and RISC prognoses showed a higher probability of death for patients in group 1 (Table 3). The RISC prognosis is determined by values collected in the hospital, including hemoglobin concentration, prothrombin ratio, and transfused pRBCs [21]. However, the prehospital administered volume directly influences these values. No significant difference was shown with regard to the likelihood of mass transfusion (Table 3). The mortality rate was significantly higher in group 1 (20.8%) than in group 2 (18.1%) or in group 3 (15.9%) (Table 3). This increased mortality rate (approximately 2%) was observed within 24 hours after admission to the hospital (Table 3).

## 4. Discussion

Regarding the emergency treatment time after severe trauma, the following principal questions must be considered: What are the major causes of prolonged emergency treatment times? Which emergency measures are already needed in the prehospital setting and must not be transferred to hospital, even if they are time-consuming?

The individual doctor beliefs in optimal scene time alone might not be the only influence in a retrospective study based on registry data. Additionally, variables such as the distance to the hospital (e.g., in a more rural environment) may have a considerable impact. Accordingly, Kleber et al. demonstrated in their study that emergency treatment time in a big city was the shortest when compared to a small town [26]. However, in their study, this had no impact on lethality and patient outcome, respectively. Due to the matching criteria that we selected in our study (e.g., rescue helicopter/ground-based transportation), and based on the large amount of 4,617 patients per group, one can assume a statistically similar distribution of urban and rural accident patients across all groups. Furthermore, it must be considered that—in isolated cases—extended rescue times may also occur in cities (e.g., in cases of technically elaborate rescue procedures).

The mechanism of the accident may also play a role. For example, emergency treatment times will be longer in cases where the technical rescue process is prolonged (i.e., when patients must be “cut out” of a wrecked vehicle). The major purpose of this study was to shed new light on factors that account for prolonged emergency treatment times in critically injured patients. However, more prospective studies are needed to further clarify this question. Based on an anonymized registry, definitive conclusions are almost impossible to draw since it is not possible to trace back to individual cases. This particular study benefits from its large sample population. It is noteworthy that the heterogeneity of the patient population would not have allowed for comparable sample sizes in a prospective study design. Moreover, unequal random patient distributions are compensated by this large sample size.

With regard to the second question, our study shows that the number of prehospital measures (e.g., chest tube insertion) correlated with increasing emergency treatment time. These measures commonly delay definitive patient care in a hospital. This is in line with recent observations by Wyen et al. Their group analyzed factors that increase prehospital emergency treatment time and concluded that these measures should be challenged and reevaluated with regard to their efficacy and necessity [27]. Although the complexity of injury patterns and related individual patient conditions does not allow standardized treatment according to comprehensive protocols, there is a general consensus that critical injuries with potentially life-threatening consequences (e.g., tension pneumothorax) must be managed within the prehospital phase even when this is time-consuming. In particular, tension pneumothorax is a condition that must be treated, and any posttrauma casualty must be prevented [2, 28]. Another study of registry data draws similar conclusions. It was shown that prehospital measures with associated extension of rescue time may improve mortality of severely injured patients and that there is no need to relocate measures into a hospital as long as they serve acute patient care [26]. This study also concludes that such measures at the accident site cannot always follow applicable protocols, but must be selected individually.

Even generally accepted principles for treating penetrating traumas cannot be transferred 1:1 to patients with blunt trauma. This, for example, applies to clamshell thoracotomy. This measure has significance in the prehospital treatment of penetrating traumas. Nonetheless, no final recommendation can be given for patients with blunt trauma, due to the current lack of evidence, although a current study from the Netherlands postulates an advantage for patients with blunt trauma [29]. Prehospital volume administration for the maintenance of sufficient blood pressure levels, i.e., cerebral perfusion pressure after isolated TBI, would be another example for different procedures in patients with blunt trauma. Target blood pressure values in patients with isolated TBI would have negative impacts on mortality in case of penetrating trauma. Nonetheless, in patients with blunt trauma, it appears to be of principal advantage when prehospital interventions are limited to stabilization of the cardiovascular and pulmonary systems to ensure rapid patient transfer to a level one trauma center [3]. This particularly applies to patients with severe TBI. Nevertheless, the nature of this study design does not allow us to distinguish whether prolonged emergency treatment time was caused by vital interventions or if prolonged emergency treatment was an independent risk factor.

It must be noted that emergency treatment times increased significantly in our study, if patients had to be sedated. As sedation is part of emergency anesthesia in conscious patients, it must be assumed that the percentage of patients with prehospital intubation (who were not initially unconscious) increases with increasing emergency treatment times. Based on the retrospective design of this study, it is only possible to suggest potential associations, and the question as to why a specific patient in group three was sedated and whether indications were given in all cases (according to current guidelines) cannot be answered conclusively [30]. In this context, recent studies have demonstrated worse outcomes in intubated patients without clear indication [31, 32]. For example, if sedation was applied due to the aggressive behavior of the patient, this may also result in posttrauma outcome worsening, as shown in an American study by Muakkassa et al. [33]. It must be emphasized, as already mentioned before, that measures during the prehospital phase almost always do increase rescue time but may still be necessary (e.g., intubation in the event of apnoea) and, thus, their implementation is mandatory.

An important insight from our study is that the emergency treatment time was increased across all study groups. The time spent getting to the accident site, the time spent at the accident site, and the time until hospital admission increased between the groups. This is not exclusively attributable to the time spent at the accident site. Nonetheless, the time at the accident site represented the major part (in patients with similar severity of injuries according to ISS). Since the rescue resource was a matching criterion, it is not possible to postulate whether a rescue helicopter would have reduced prehospital time, particularly since registry data do not identify whether the rescue helicopter was ordered initially or called for by a ground-based emergency physician from the accident site at a later stage (e.g., due to the injury pattern). However, in the current literature, the employment of rescue helicopters is commonly associated with an increase in emergency treatment time [34]. Nevertheless, in a German study, Andruszkow et al. observed a survival benefit related to rescue helicopter transfer [35]. A possible reason could be that rescue helicopters teams (HEMS) usually have a greater experience in prehospital trauma scenarios, especially in the rural setting. For example, Andruszkow et al. did not find preventable trauma deaths when trauma patients were treated by HEMS crew [35].

In strong contrast to existing data, our study showed an increase in mortality associated with reduced emergency treatment times. It is of note that, in the current literature, short emergency treatment times (i.e., rapid transfer to a hospital for definite care) are commonly associated with a reduction in mortality [3, 4, 6]. For example, in a recent US study, Swaroop et al. demonstrated that short emergency treatment time is associated with survival benefit after penetrating thoracic trauma [36]. In contrast, the study by Fuller et al. suggested that patients with traumatic brain injury experience better outcomes when admitted to specialized hospitals for treatment [37]. As mentioned before, it is not possible in a retrospective study to answer the question conclusively as to why the emergency treatment time was prolonged in specific cases. However, the results indicate that the majority of the most-severely injured patients in critical condition were found in the short emergency treatment time group. Accordingly, the proportions of patients with GCS scores ≤8, of resuscitated patients (who were either resuscitated in a prehospital or in a hospital setting), and of patients who were admitted to the hospital with hemorrhagic shock were highest in this group. Nevertheless, it must be considered that the TR-DGU® comprises only patients who were admitted to a hospital. Patients who were deceased at the accident site or died on their way to the hospital are not available for analysis. This represents a selection bias in this patient population that could provide an additional explanation for the better outcome in group 3, since the number of patients who died at the accident site or on their way to the hospital may be higher in this group. This assumption is also supported by the fact that the outcome in group 3 was better when the groups were compared by RISC prognosis. In their study, Kleber et al. concluded in a comparable trial that the selection process due to the study design prevents conclusive data interpretation [26].

In addition to the potential selection bias in group 3, this management can also lead to quicker identification of the patients' injury severity by the EMS team members, thus allowing more precise and faster therapy at the accident site. Consequently, severely injured patients are more rapidly hospitalized. Thus, patients who would have otherwise died at the accident site were admitted to the hospital and enrolled in the trauma registry. Similar results were demonstrated by Ball et al. in patients with abdominal injuries, where the number of patients who reached the hospital increased with reduced emergency treatment times [38]. In our study, this could particularly apply to group 1, which, e.g., comprised significant more patients with prehospital CPR. These patients possibly had injuries they could not survive, but reached the hospital due to short rescue times, and, thus, were recorded in the TraumaRegister DGU. This is also suggested by the larger number of surgical emergency procedures and the mortality rates after 1 and 24 hours, respectively. The lower actual mortality in contrast to a higher statistical mortality risk (according to RISC and TRISS) in this group may be due to shorter rescue times. Another indicator for a more rapid assessment of injury severity by EMS staff in this group can be possibly seen when considering treatment times at the accident site. Treatment times represent the major part of total time figures across all groups but were shortest when compared between groups 1-3, despite identical injury severity (ISS). In principle, one can assume that emergency therapy at the accident site is solely based on the patient's injury and, thus, should be similar in all groups.

Apparently, the dependence on the emergency team members' level of education and experience that was postulated by Oestern in the late 1990s has become less important due to the presence of extensive and nationwide training programs [39]. It is likely that emergency team members are more aware of the essential necessity of rapid diagnosis and therapy (which has also been called for in the current literature) as a result of training courses such as the Pre Hospital Life Support (PHTLS®) course and are paying more attention to the time factor. Gao et al. emphasized in their study that rapid diagnosis and therapy are associated with improved outcomes [1]. However, in our study, it was only possible to suggest potential associations.

Reduced emergency treatment time in critical patients following severe trauma might also be attributable to an improved overall infrastructure. German trauma networks that were established and trained on a nationwide basis contribute substantially to the reduction of emergency treatment times. This benefit has not only been observed in Germany but also in the US and in other countries [40–42].

### 4.1. Limitations

(1) Prolonged prehospital time may result from delayed alerting of the EMS, from time-consuming technical rescue processes (e.g., wrecked vehicles) or from ordering a rescue helicopter at a later stage (i.e., by a ground-based emergency physician). Based on the anonymized TR-DGU® data, this question cannot be answered conclusively.

(2) With regard to the coagulation status analysis, it must be noted that prothrombin time, prothrombin ratio, and platelet counts are the only parameters documented and available in the TraumaRegister DGU®. The registry did not document other laboratory values that might have been of interest for coagulation (e.g., fibrinogen and protein C).

(3) The matched-triplet analysis is dependent on the quality of the matching criteria. When patients were matched, not all the patients in the TraumaRegister DGU® were included since patients without a “partner” were not retained in the analyses. The advantage of comparing patients in the matched-triplet analysis, however, is the fact that small differences can be detected.

(4) The TraumaRegister DGU® only enrolls patients who were admitted to the hospital alive. No statements can be made with regard to patients who were deceased at the accident site or died during transportation. This represents some type of selection to a certain degree.

(5) TRISS calculations could be performed for only 46% of the participating trauma centers, whereas the RISC analysis was possible for 88% of the cases. Thus, as TRISS calculations could not be performed for the majority of trauma cases, the data might be biased. However, this suggests that calculation of the RISC is easier than TRISS quantification. This might be explained by the fact that RISC does not determine the prehospital respiratory rate, which is only documented in 60% of cases by physicians at the accident site.

(6) Based on the anonymized data of the TraumaRegister, it cannot be clarified in individual cases, as to whether all prehospital therapies were indicated according to current guidelines.

Since retrospective data analysis was performed, only associations, not causality, can be ascribed to the results shown here.

## 5. Conclusions

Our results suggest that survival after severe trauma is not only a matter of short rescue time but more a matter of well-used rescue time including performance of vital measures already in the prehospital setting. This also includes that rescue teams identify the severity of injuries more rapidly in the most-severely injured patients in critical condition than in less-severely injured patients and plan their interventions accordingly. Therefore, patients who would have otherwise died at the accident site survive and are admitted to the hospital. All in all, it is important to find a balance between both a prompt and individually adapted and potentially time-consuming therapy, in order to eventually create a positive impact with regard to the outcome of most-severely injured patients.

## Figures and Tables

**Table 1:** Demographic and clinical data of severely injured patients treated prior to hospitalization.

	Group 1	Group 2	Group 3	All patients	p-values
Patients (n)	4617	4617	4617	13851	
Age in years (MV, SD)	45.8 ± 19.9	45.6 ± 20	45.3 ± 20.3	45.6 ± 20.1	0.30
Age ≥60 years (%)	25.9	25.9	25.7	25.8	0.90
Male (%)	71.8	74.2	72.4	72.8	0.03
Glasgow Coma Scale ≤8 (%)	36.6	33.5	30.3	33.4	≤ 0.001
Injury Severity Score (MV, SD)	29.2 ± 12.8	28.6 ± 12.0	28.2 ± 11.5	28.7 ± 12.1	0.100
Traffic accident (%)	62.7	62.1	63.2	62.6	0.6
AIS head ≥3 (%)	55.7	55.7	55.7	55.7	1
Isolated TBI (%)	11.8	11.4	12.1	11.7	0.6
AIS thorax ≥3 (%)	59.3	59.3	59.3	59.3	1.00
AIS abdomen ≥3 (%)	17.6	17.6	17.6	17.6	1.00
AIS extremities including pelvis ≥3 (%)	35.7	35.7	35.7	35.7	1.00

Values are shown as the mean (MV), standard deviation (SD) or % of the group. AIS, Abbreviated Injury Scale. TBI, traumatic brain injury.

**Table:** (a) Group-specific patient data regarding emergency treatment time and vital signs

	Group 1	Group 2	Group 3	All patients	p-values
Arrival of emergency doctor after accident (in min.; mean, SD)	11.9 ± 5.6	16.6 ± 8.2	28.3 ± 24.7	19.1 ± 17.0	≤ 0.001
Time spent by emergency doctor at accident site (in min.; mean, SD)	19.3 ± 7.6	29.5 ± 10.4	45.7 ± 21.3	31.9 ± 18.4	≤ 0.001
Time from accident site to A&E (in min.; mean, SD)	11.0 ± 5.5	17.4 ± 8.0	27.7 ± 17.3	19.1 ± 13.5	≤ 0.001
Total prehospital time (in min.; mean, SD)	40.1 ± 8.3	62.9 ± 6.9	101.1 ± 26.0	68.0 ± 29.9	≤ 0.001
BP at accident site (mmHg; mean, SD)	119.3 ± 37.9	120.6 ± 36.8	121.0± 34.8	120.3 ± 36.5	0.53
BP at admission to hospital (mm Hg; mean, SD)	121.2 ± 33.9	122.5 ± 33.3	123.9 ± 30.3	122.5 ± 32.5	0.24
BP at accident site <90 mm HG (%)	18.4	18.4	18.4	18.4	1
BP <90 mm HG in the emergency department (%)	14.7	14.1	12.6	13.8	0.01
Heart rate at accident site (sec.; mean, SD)	91.4 ± 26	92.4 ± 25.1	93.0 ± 24.2	92.3 ± 25.1	0.24
Heart rate upon admission to hospital (sec.; mean, SD)	88.5 ± 23.5	89.8 ± 21.9	89.5 ± 20.8	89.3 ± 22.1	0.71

Values are shown as the mean (MV), standard deviation (SD) or % of the group. BP, blood pressure.

**Table:** (b) Group-specific patient data regarding laboratory values in-hospital

	Group 1	Group 2	Group 3	All patients	p-values
Hb upon admission to hospital (mg/dl; mean, SD)	12.0 ± 2.8	11.9 ± 2.8	11.9 ± 2.7	11.9 ± 2.8	0.04
Prothrombin ratio (%) in hospital	80.7 ± 23.0	80.0 ± 23.1	79.6 ± 23.1	80.1 ± 23.1	0.03
Platelet count/nl upon admission to hospital (mean, SD)	209140 ± 80468	204319 ± 75993	202959 ± 80165	205399 ± 78896	≤ 0.001
Prothrombin time in hospital (sec., mean, SD)	35.2 ± 21.2	34.9 ± 22.4	34.1 ± 18.9	34.7 ± 20.9	0.09
Base excess in hospital (mean, SD)	-3.2 ± 4.9	-2.8 ± 4.9	-2.7 ± 4.6	-2.9 ± 4.8	0.03
TASH (mean, SD)	8.4 ± 15.5	8.4 ± 15.1	7.9 ± 14.3	8.2 ± 14.9	0.87

Values are shown as the mean, standard deviation (SD) or % of the group. Hb, hemoglobin; TASH, Trauma Associated Severe Hemorrhage.

**Table:** (c) Group-specific patient data regarding prehospital and in-hospital therapies

	Group 1	Group 2	Group 3	All patients	p-values
Fluid replaced prehospital (in ml; mean, SD)	1135 ± 950.2	1280 ± 934.7	1461 ± 1095.8	1291.8 ± 1004.9	≤ 0.001
Fluid replaced in the emergency department (in ml; mean, SD)	2234.9 ± 3080.2	2203 ± 2287.1	2360.5 ± 2599.5	2266.4 ± 2667.9	≤ 0.001
Fluid replaced prehospital (%)	93.8	95.4	95.7	95.0	≤ 0.001
Prehospital use of catecholamines (%)	9.0	9.2	9.1	9.1	0.97
Transfusions of pRBC (%) in hospital	24.1	25.6	25.1	24.9	0.21
Units of pRBC in hospital (mean, SD)	1.9 ± 5.5	1.9 ± 5.4	1.8 ± 5.2	1.9 ± 5.3	0.27
Units of fresh-frozen plasma in hospital (mean, SD)	1.0 ± 4.2	1.1 ± 3.9	1.0 ± 3.8	1.0 ± 4.0	0.21
Prehospital intubation (%)	52.7	52.7	52.7	52.7	1.00
Intubation in hospital (%)	56.4	55.6	57.0	56.3	0.12
Prehospital chest tube (%)	5.2	5.8	6.9	6.0	0.01
Chest tube in hospital (%)	25.4	23.0	22.1	23.5	0.003
Prehospital CPR (%)	5.2	4.0	3.0	4.1	≤ 0.001
CPR in hospital (%)	5.9	4.8	3.3	4.6	≤ 0.001
Prehospital sedation (%)	76.9	82.0	84.2	81.1	≤ 0.001
MSCT in hospital (%)	68.0	68.2	70.0	0.12	0.100

Values are shown as the mean, standard deviation (SD) or % of the group. pRBC, packed red blood cells; CPR, cardiopulmonary resuscitation; MSCT, multislice computed tomography.

**Table 3:** Clinical course and outcome of patients with short, intermediate, or long emergency treatment times after trauma.

	Group 1	Group 2	Group 3	All patients	p-values
Early SURG (%)	6.7	5.8	5.2	5.9	0.02
Stay in intensive care unit (%)	93.4	94.3	94.8	94.2	0.02
Days in the intensive care unit (mean, SD)	11.3 ± 13.9	11.3 ± 13.4	11.4 ± 13.4	11.3 ± 13.6	0.01
Days intubated (mean, SD)	7.0 ± 11.5	7.0 ± 10.8	7.1 ± 11.3	7.0 ± 11.2	0.06
Organ failure (%)	49.1	47.8	46.4	47.7	0.10
Multiple organ failure (%)	31.6	29.6	28.2	29.8	0.01
Sepsis (%)	9.3	8.6	10.0	9.3	0.14
RISC prognosis (mean, SD)	21.9 ± 29.3	20.5 ± 28	18.7 ± 25.7	20.4 ± 27.7	≤ 0.001
TRISS prognosis (mean, SD)	24.9 ± 32.2	22.2 ± 30.1	20.4 ± 28.4	22.6 ± 30.3	≤ 0.001
Died in hospital (%)	20.4	18.1	15.9	18.1	≤ 0.001
Died within the first hour (%)	2.3	1.3	1.0	1.5	≤ 0.001
Died within the first 24 hours (%)	12.0	9.7	8.2	10.0	≤ 0.001
Days of hospitalization (mean, SD)	24.6 ± 25	25.7 ± 26.8	25.6 ± 24.5	25.3 ± 25.4	≤ 0.001

Values are shown as the mean, standard deviation (SD) or % for the group. ED, emergency department; SURG, surgery; ICU, intensive care unit; RISC, Revised Injury Severity Classification; TRISS, Trauma and Injury Severity Score.

## Data Availability

All data generated or analyzed during this study are included in the article.
